# New choreographies of inequalities in reproduction: an overview of assisted reproduction market

**DOI:** 10.5935/1518-0557.20230026

**Published:** 2023

**Authors:** Sandra Mara Garcia

**Affiliations:** 1 Senior Researcher at CEBRAP - Brazilian Center for Analysis and Planning, São Paulo, Brazil

**Keywords:** low fertility, infertility, assisted reproduction, reproductive technologies, transnational reproduction, public policies

## Abstract

Brazil follows the trend of countries that went from high fertility to below replacement level; in many countries, fertility rates continue to fall, often to levels well below population replacement, especially in Europe and Eastern Asia. Since 2006, Brazil has presented rates below the population replacement level, with regional variations. The shift to a pattern of late motherhood is central to understanding this phenomenon, as well as the increased use of reproductive technologies and the global market for assisted reproduction. Demand for services based on Assisted Reproductive Technologies (ART) has increased in European countries and the United States. Also, in Brazil, there is a growing demand for assisted reproduction services, which private clinics offer at a significantly high cost. This article provides an overview of these issues. It raises new questions and dimensions of analysis by problematizing the socio-demographic, legal, and ethical aspects of assisted reproduction, which need to be explored in future population studies.

## INTRODUCTION

Fecundity, as a human ability, is profoundly significant for all societies, which depend on it for their members to reproduce. From a demographic perspective, we have witnessed relevant changes in Western societies in recent decades, such as female emancipation and changes in the age composition of populations and family structures (te Velde, 2011). These collective transformations affected the symbolic models that govern the identification of subjects, particularly sexual and gender identities and, above all, those related to filiation, maternity, and paternity (Tort, 2001).

Studies in the fertility field - one of the components of demographic dynamics - reveal large-scale declines in fertility rates (average number of children that a woman has throughout her reproductive life) from the 1960s, especially in high and middle-income societies considered more developed.

Changes from high to low levels of mortality and fertility have been universally experienced, and it is one of the most striking features of the demographic transition. This process occurs at a different pace between countries due to their various social, economic, and institutional contexts and interactions (Willekens, 2015). Countries that show fertility rates below the population replacement level^1^ range from low fertility, such as China (1.7), the USA (1.6), the UK (1.6), and Portugal (1.4), to very low fertility, such as Greece (1.3) Japan (1.3), Italy (1.2), Spain (1.2), Ukraine (1.2), Singapore (1.1) and South Korea (0.9) (World Bank, 2020). In Brazil, the decline in fertility that began in the mid-1960s followed its downward course, from 6.3 children per woman to 1.7 in 2018, although this figure varies by region, income, education, and race.

The determining factors for the decline in fertility in the last five decades are related to processes of extremely significant sociocultural, economic, political, scientific, and technological transformations, which have contributed to re-signifying the relationships between genders, as well as perceptions, preferences, and attitudes towards the size and type of family arrangements. Some of the consequences of these processes in the dynamics of fertility were the increase in the average age of women in their first union and the birth of their first child, the increasing postponement of motherhood until after the age of 30, and the increase in single-parent families. Coutinho & Golgher (2018) used data from the DHS from 1986, 1996 and the PNDS from 2006 to estimate competitive preferences for women who did not realize the desired children reported. The authors identified that wealthier and more educated women might face factors that compete more effectively with motherhood, such as careers and long-term education.

Among the characteristics that contributed to the postponement of motherhood, the following stand out: the increase in female education, the prioritization of building a career, particularly involuntary childless among women of higher socioeconomic and educational levels, political and structural gendered inequalities - especially in private settings, when it comes to household and childrearing and the occurrence of new unions combined with the desire to have children with new partners. Such a transformation would not have been possible without access to contraceptives, which made it possible to separate sex from reproduction and postpone motherhood until “the right time” to have the first child (van de Kaa, 2011). A study carried out by Miranda-Ribeiro *et al*. (2019) based on data from the Brazilian Census shows a scenario of postponement of the first child and an increase in the proportion of women who end the reproductive period without children, despite regional variations.

However, postponing reproduction to “the right time” may not align with the female “biological clock.” The association between this postponement and the increased percentage of involuntary childlessness among women is already widely known, either because of the difficulty in getting pregnant due to the decline in female fecundity after the age of 35 or because of not being able to carry the pregnancy to term due to recurrent miscarriages, resulting in smaller families than initially desired (Sobotka & Beaujouan, 2018).

However, there is a necessary distinction between difficulty becoming pregnant due to declining fecundity and infertility as a pathological condition. The World Health Organization (WHO) recognizes this condition as a severe global health problem^.^ It has several associated causes that affect both sexes, such as cystic fibrosis, infections such as chlamydia and gonorrhea, and systemic diseases. Regarding women, polycystic ovary syndrome, premature ovarian failure, endometriosis, and uterine fibroids stand out. As for men, there are testicular and post-testicular deficiencies. The decline in semen over the years is another likely associated factor. It is estimated that infertility affects 8-15% of couples of childbearing age worldwide, recording an increasing trend due to age (WHO, 1999). However, no clinical and epidemiological data for Brazil indicate the magnitude of the problem, its features, and its social impacts.

The National Survey on Demography and Health of Women and Children (PNDS) 2006 (Berquó *et al*., 2009) provides sociodemographic data on self-reported infertility or difficulty getting pregnant in the female population of childbearing age. These data revealed that of the 10,575 women aged 15 to 49, 7% declared themselves infertile or having difficulty conceiving, and 21% reported being sterilized. Almost half of those who claimed themselves infertile stopped seeking help from health services, most of them being black women (59%). In addition, of the sterilized women, 12% regret it, and of these women, about 70% declared that they wanted another child (Garcia & Koyama, 2014). It was impossible to determine through the survey whether the information obtained was based on a medical diagnosis.

However, infertility needs to be diagnosed so treatment is possible. The recommendations are drug therapies, corrective surgery, and medically assisted reproduction techniques. Commonly neglected in developing countries, where public policies are incipient, infertility has social, economic, and psychological consequences on couples. In general terms, it can be said that the causes of infertility are well divided: approximately 35% of cases are related to female factors and 35% to male factors. Another 20% is associated with the couple’s combined infertility, and 10% is due to unknown causes (de Santiago & Polanski, 2022). Regarding female fecundity, the most critical component of its steady decline is the woman’s age. From 30 onwards, there is a decrease in the number and quality of oocytes (Baird *et al*., 2005; Broekmans *et al*., 2007; Olsen, 1990). In turn, male fertility is also affected by age; although sperm production is constant, increasing age impacts semen quality and can decrease the chances of pregnancy. Alternatively, male infertility can still be associated with problems during pregnancy, such as miscarriages, preterm deliveries, stillbirths, and possible risks of genetic malformations (de La Rochebrochard *et al*., 2006; Sartorius & Nieschlag, 2010).

In this sense, although the biological limits for women are more evident and further investigated, the “biological clock” also affects men. Nevertheless, research on reproductive capacity has focused much more on women, as evidenced by the extensive research on infertility and comorbidities in women who have delayed pregnancy beyond age 35. Few studies have explored the risks of late parenthood on children’s health, mistakenly suggesting that women are held more accountable than men for pregnancy failure or even for adverse health outcomes in live births (Phillips *et al*., 2019; Hens, 2017).

Sociocultural factors also contribute to the fact that the focus of investigations on infertility falls mainly on the female body. In population studies, the role of men in reproduction and reproductive planning has long been neglected (Garcia, 2006). Only recently have demographic surveys (DHS)^2^ included male-specific questionnaires about contraception.

### Assisted reproduction: a transnational and unequal market

Given the issues discussed here, a growing fraction of women resort to assisted reproductive technologies (ART) to fulfill their reproductive desire. Regardless of the enthusiastic or alarmist considerations that surround it, assisted reproduction was a milestone of the technological revolution in the field of Biomedicine and is considered strategic technology given its potential to transform future life (Garcia & Bellamy, 2015; Corrêa & Loyola, 1999; 2015; Corrêa, 2001).

This mostly private market consists of all products used in the procedures, such as equipment, devices, reagents and pharmaceuticals, and laboratory and medical services to carry out the “fertilization cycle.” Depending on the case, the procedures may include cryopreservation of gametes and embryos, donation of genetic material (semen, oocytes, and embryos), and sometimes surrogate pregnancy, known as “surrogacy.” Forty years ago, a woman could not conceive a child that was not genetically related to, and conceived by her. The consequences of this technological revolution in reproduction are far-reaching, significantly impacting family structures. It opens new ways for fulfilling the desire for motherhood/fatherhood for hetero and homosexual couples or women and men alone through oocytes and semen from donors and surrogate mothers. Furthermore, children born into these families may have half-siblings anywhere in a country or even outside of it. However, they are unlikely to have any contact (Spar, 2006).

Demand for ART-based services has grown significantly in European countries and the United States and has expanded to Asia and the Middle East. In Europe, 1.5% of births are already via ART; in Denmark, they represent 8% of births. As for the U.S., 1.8% of births in 2018 were through ART, a three-billion-dollar-per-year market. In China, after the end of the one-child policy, this market expanded, with transactions amounting to US$ 1.1 billion in infertility treatments in 2016 alone. Russia and Ukraine are the fastest growing markets due to their low fertility rate, looser regulation, and lower costs; in 2018, these countries transacted around US$ 7 billion, with a growth forecast of 11% between 2019 and 2029. Estimates show that this global market should total US$ 45.4 billion by 2025 (Grand View Research, 2018). Even with this expansion, access is unequal due to the high costs of highly complex procedures. Access to ART differs globally in terms of availability and affordability, particularly in less developed countries and among different socio-cultural contexts. In a few countries, state policies that make them economically accessible through legislative actions are in place. Incipient initiatives led by physicians are under development, particularly in Africa, to provide more accessible IVF (*In Vitro Fertilization*) to the population (Inhorn & Patrizio, 2015). In other words, the logic and vision of the market in medicalizing procreation prevails.

“How much is a baby worth?” Estimates indicate that the U.S. is where ART services are the most expensive. According to Schurr (2018), “for US$50,000, it is possible to provide four cycles of IVF in a North American clinic or ten cycles in a Ukrainian clinic” or pay for surrogacy in Mexico, which costs three times more in the U.S.

In this private and socially excluded model, a new market feature has taken shape among large hi-tech companies in the U.S., based on incentive policies to retain female labor. Facebook, Google, and Apple propose to cover the costs of oocyte cryopreservation as part of the benefits for young women. Regardless of the companies’ good intentions, these measures reveal a deeper aspect of the complex relationships between women’s reproductive autonomy and the labor market, which necessarily involve much more complex social, political, and cultural issues to be resolved (Mertes, 2015). Oocyte cryopreservation may offer companies another way to deny women’s adequate parental leave. In contrast, successful conception with stored oocytes may still be low for women who undergo this procedure after age 35 (Waldby, 2019).

In addition to economic barriers, other cultural and regulatory barriers restrict the circulation of genetic material and the practice of “surrogacy.” Some technologies and services are legal only in certain countries; elsewhere, access to specific populations, such as single people or same-sex couples, is prohibited. This generates mobility in search of treatment. This “reproductive tourism” or “transnational tourism” usually originates from countries with more restrictive and high-cost legislation to countries with less moral restrictions and lower costs, such as Ukraine.

The global surrogacy market brings ethical dilemmas over state responsibility regarding its acceptance and regulation, dividing countries (and internally), leading to wide variations in how commercial surrogacy arrangements are regulated. Those that prohibit it altogether, others that govern all aspects of surrogacy, and those where it is partially legalized. The problem exists between the market and the country’s legislators who seek to follow moral customs and appropriate socio-cultural norms, including the religious environment. Currently, “surrogacy” is prohibited in Germany, Austria, Denmark, Finland, Italy, Spain, Norway, Switzerland, Serbia, Pakistan, and Malaysia. On the other hand, it is allowed in Egypt, Israel, South Africa, Ukraine, and Russia. India used to have the most significant market share; but recently, in 2018, the country banned it for foreigners. It is worth mentioning that the work of France Twine (2015), whose comparative analysis of this sector in developing countries such as India, China, and Ukraine, as well as the United States and Israel, gives a critical and complex view of a form of gender work. According to the author, such gender work is being outsourced and it is a growing segment of the medical tourism industry.

Likewise, the oocyte market varies from country to country and can meet different demands. It is entirely illegal in Italy, Germany, and Austria; whereas it is legal, anonymous, and without compensation in France; allowed, non-anonymous, and without payment in Canada; legal, unknown, and with financial compensation in Spain, Czech Republic, South Africa, and Greece; allowed, non-anonymous and with monetary compensation in the UK. In this regard, the U.S. emerges as the most diversified market since it is legal, anonymity is optional and financial compensation is allowed (Waldby, 2019).

However, in terms of size, Europe is considered the largest reproductive market due to low fertility rates, the growth of infertility, more exceptional knowledge about technological advances for infertility treatment, and the presence of several government initiatives. Furthermore, regarding regulatory aspects, Europe has been expanding the regulation of reproductive technologies since 2009, and all countries now have some form of law in this regard. Only half of the European countries allow single women to use ART (Präg & Mills, 2017). Spain allows access to assisted reproduction for female couples, limiting access to male couples. Other countries allow it for single women but not for female couples; such as Italy, Finland, Greece, Cyprus, Malta, Bulgaria, and Poland. In contrast, France, in 2018, changed its law by allowing access for single women and female couples. So far, Denmark stands out for the more inclusive nature of its regulation, given that its public health service offers three cycles of IVF, and access to IVF is allowed for all women, regardless of their marital status or sexual orientation.

However, the trend is to review the “surrogacy” permission among European countries, especially as they present complex problems to be solved, such as the legal status of children born in other countries. This discussion is ongoing, and for now, that is how the limits imposed by each country’s social, cultural, economic, and political forces are set up. Moreover, it may undergo new limitations depending on the forces influencing the public and political debate.

### Aspects of the Brazilian assisted reproduction market

Since the first birth by in vitro fertilization in Brazil in 1984, much has been researched on assisted reproduction in Brazil and its ethical, legal, and social aspects, but much remains to be investigated. Given the privatizing logic that marks ART in the country, problems associated with infertility await due attention and prioritization (Corrêa & Loyola, 2015; Garcia & Bellamy, 2015; Garcia *et al*., 2013; Garcia & Ramirez, 2014; Alfano, 2014; Samrsla *et al*., 2007). In this sense, the association between untreated sexually transmitted infections (STIs) in women; and most cases of tubal factor infertility is widely documented (Ombelet, 2011; Paavonen & Eggert-Kruse, 1999). Although scarce, some studies investigated the association between Chlamydia trachomatis infection and infertility in Brazil (Approbato, 2012; Lavorato *et al*., 2015; de Assis *et al*., 2021). Fernandes *et al*. (2014) evaluated the prevalence of tubal obstruction caused by chlamydia and gonorrhea in women treated in the public reproduction service in the Center-West region between January 2009 and December 2012. This study showed that tubal obstruction (41.2%) was the most frequent infertility factor in the population analyzed. More than half of these patients (56.8%) had infections caused by chlamydia and gonorrhea. This is because they are asymptomatic diseases often detected late, thus impairing treatment; some women only find out about STIs when they are pregnant during prenatal care. Therefore, women who do not become pregnant may discover these infections at a worse stage. The prevalence of these diseases among the sexually active population of reproductive age increases the importance of incorporating effective screening for early detection of sexually transmitted infections for appropriate treatment and as part of routine reproductive health care.

In Brazil, there is a growing demand for assisted reproduction services, which private clinics offer at a high cost. Each fertilization cycle can range from R$15,000.00 to R$30,000.00, depending on the benefits and treatments included^3^. There are approximately 180 assisted reproduction clinics (BCTGs)^4^, which are unequally distributed across the country, with 57% concentrated in the Southeast. Only ten of BCTGs are public services. Within the scope of these services, treatment is limited in terms of the techniques offered, and access depends on a waiting list, which can take up to four years. (Garcia & Bellamy, 2015; Garcia *et al*., 2012; Makuch *et al*., 2011; Corrêa & Loyola, 2015).

Research data on assisted reproduction in Brazil carried out by Garcia & Bellamy (2015)^5^ showed that in the absence of a specific law on this matter, the Federal Council of Medicine (CFM) has been playing the role of the disciplinary body since 1992, by preparing technical standards for the use of reproductive techniques. However, they have no legal force. In turn, the Brazilian Health Regulatory Agency (ANVISA), as a regulatory and supervisory body for the BCTGs, requires annual information to be sent to the SisEmbrio^6^ on the types of procedures performed: the number of cycles, the number of oocytes collected, the number of embryos produced, implanted, cryopreserved, and discarded. Data on pregnancies and live births are not part of SisEmbrio’s information.

Since 2012, these records have shown a steady growth of cycles performed and embryos produced, primarily concentrated in the southeast and the country’s south. In 2012, 21,074 fertilization cycles were performed, against 46,010 in 2021(Anvisa, 2012; 2022), an increase of almost 120% in 10 years. As for the freezing of embryos, considering this same time interval, 32,181 embryos were frozen in 2012, against 202,875 in 2022 (Anvisa, 2012; 2022), an increase of 530%. On the other hand, we saw an increase in the flow of genetic material (semen and oocytes) to Brazil. Data provided by ANVISA itself show an impressive growth between 2011 and 2017 in semen imports, from 16 samples to 860, mainly of Caucasian origin, coming from the United States^7^. The leading importers are, in this order: single women, heterosexual couples, and female couples. There was also a considerable increase in oocyte imports between 2015 (22) and 2017 (321). Previous data are not available in Anvisa Reports (Anvisa, 2018). This flow is much higher if we consider Brazil’s clandestine oocyte market. Consequently, its size is unknown. A quick internet search reveals the supply of oocytes by Brazilian women in Brazil and other countries. That is, this market escapes Anvisa’s inspection and disagrees with the rules established by the CFM on anonymity and the prohibition of trade in gametes.

Likewise, the offer of “surrogacy” for financial reasons has been widely publicized on the Internet, despite not having normative support from the CFM or the Brazilian legal system. Until 2021, the technical standards defined by the CFM said that only relatives up to the fourth degree of those involved could deliver the uterus to the surrogate maternity. In September 2022, the CFM updated the surrogacy rules, allowing women not related to the couple or one of the partners, through exceptionality authorization, to apply to the jurisdiction’s Regional Council of Medicine (CRM). Financial compensation remains prohibited by this regulation (CFM Resolution No. 2.320, 2022).

As for reproductive tourism as a destination, Brazil has been on the route for a long time due to the demand for infertility treatment by foreign couples at assisted reproduction clinics. According to Machin *et al*. (2018), information from 84 clinics that responded to an online survey showed a growing increase in upper-middle-class Angolan women who, during the treatment process, established residence in Brazil, along with other women in the same situation. Many extended their stay in Brazil for months after giving birth, having secured medical care through the Brazilian Unified Healthcare System-SUS Machin *et al*. (2018). Assisted reproduction services remain inaccessible in Angola and other sub-Saharan African countries due to scarce health resources, reliable diagnosis, and treatment, including IVF services (Inhorn & Patrizio, 2015).

## DILEMMAS AND CHALLENGES FOR REGULATION POLICIES AND RESEARCH AGENDA IN BRAZIL

It is interesting to illustrate some of the recurring themes in the national conferences’ agendas regarding assisted reproduction that reveal social, ethical, and legal aspects pervading the practices and knowledge of this field (Garcia *et al.,* 2013; Garcia & Ramirez, 2014). Among the topics discussed, the concern with the commercialization of gametes stands out, a practice that is prohibited but exists beyond the physician’s control. Another concern is the incompatibility between the donor’s right to anonymity and the child’s right to genetic information. Supported by Article 48 of the Child and Adolescent Statute, by analogy with adoption, and Article 7 of the Universal Declaration on the Human Genome and Human Rights, to which Brazil is a signatory, children have the right to know their genetic ascent. Countries that opened secrecy lost donors, such as England and Sweden. The lack of legal validity of the contract for the temporary assignment of a uterus is also a cause for concern, even if it is carried out by the established technical standards, given the unavailability of a human body and the rights of the conceived child even more so when the world trend is for the prohibition of this procedure under any circumstances. In this sense, the recurring question is: How to avoid legal challenges in assisted reproduction?

Another issue concerns using the PGT (Preimplantation Genetic Testing)^8^ technique. It involves testing the genes of embryos created by *in vitro* fertilization. It has been recommended for recurrent miscarriage and repeated implantation failures to select embryos with unwanted genes and avoid the transmission of hereditary diseases previously identified in the family. This is under CFM regulations and the Brazilian Biosafety Law (2005)^9^. However, by allowing the sex of the embryo to be known, another concern is that it could be used to choose the sex of babies for non-medical reasons^10^, which is expressly prohibited. As a result, in May 2021, the CFM updated the ethical standards for the use of assisted reproduction techniques, clearly stating that “to avoid social sexing, the embryonic genetic study report will only inform whether the embryo is male or female in cases of sex-linked diseases or sex chromosome aneuploidies.” However, in 2022, a new Resolution was published, bringing updates to the rules for AR. Regarding the pre-implantation genetic diagnosis of embryos, it reinforces the orientation that AR techniques cannot be applied with the intention of selecting the sex, except to avoid diseases in the possible ascendant: “assisted reproduction techniques cannot be applied with the intention of selecting the sex (presence or absence of a Y chromosome) or any other biological characteristic of the child, except to avoid diseases in the possible offspring.” However, the previous determination on the complete information of the genetic report was omitted. There is no information about the reasons for this change.

A critical point of the debate is the fate of embryos considered to be surplus and that are cryopreserved in BCTGs. According to CFM rules and Anvisa resolutions, the clinics are responsible for maintenance. However, when embryos are abandoned and authorization for disposal is required, there is concern about the cost of keeping them in appropriate spaces. For how long? When can it be discarded? How can this disposal be done? Given this demand, in 2017, a new CFM resolution shortened the maximum time for maintaining embryos in clinics before removal from 5 to 3 years.

Furthermore, discussions on the regulation of assisted reproduction in the Brazilian legal system due to bills being processed in the National Congress. Bill 1184/2003 covers essential topics from a technical and bioethical point of view and has been in process since 2003. It is a setback in several aspects from what is practiced today, as it prohibits surrogacy, limits the implantation of embryos, makes the identity of donors known, and restricts access to same-sex couples and single women. The legislative discussion is still timid and threatened by the religious forces in Congress. Therefore, the fear is that a law on assisted reproduction will “imprison doctors and users in a straitjacket,” leading to restrictions and setbacks. However, the scenario indicates that such a bill will not be approved soon.

As there is the manipulation of gametes and embryos during highly complex procedures in assisted reproduction, there is a strong interest from religious sectors in vetoing any treatments involving the subject^11^ (note). Associations between assisted reproduction and abortion are recurrent, another issue susceptible to religious groups.

In the case of Brazil, despite being a secular state, 65% of the population declare themselves Catholic and 22% Evangelical (IBGE, 2010), which implies a significant number of legislators with religious influence.

In surveys of interest to religious currents, the Catholic and Evangelical benches unite, even leaving aside party differences. These two groups internally dispute the legislative power and the regulation of laws in the area of assisted reproduction (Garcia *et al*., 2012).

Without specific legislation on assisted reproduction, science and society are advancing in giant steps. The CFM has been playing the role of regulator and catalyst for the yearnings of a portion of the population that can access services. A recent study by Machin *et al*. (2020) highlights that the new CFM resolutions moved from the predominant concept of “health care” to “meeting new family configurations.”

From the point of view of the contributions of the human sciences to the topic, the discussions are vibrant and come mainly from the disciplinary fields of anthropology and bioethics. They cover transnational phenomena: globalization, stratification, exploitation, religion, biopower, and bioethics.

Initially, anthropological studies of a feminist nature were divided between positions of caution about the harms of reproductive technologies and evaluative overstatements about the “salvation of women from the need to reproduce” (Nahman, 2016). More recently, production in the area has expanded and intensified research into the connection between reproductive technologies and culture, kinship, economy, nation, race, religion, and globalization.

Many Brazilian scholars have discussed the public funding of assisted reproduction, emphasizing the importance of public health interventions and programs to address the harm associated with the involuntary absence of children (Corrêa & Loyola, 2015; Garcia & Bellamy, 2015; Makuch *et al*., 2010; 2011). These studies consider legal frameworks, such as the Family Planning Law of 1996 (Law 9263, Article 226), which indicates the State’s duty to “Assist with all methods and techniques for contraception and conception,” and the Program of Action of the Cairo Conference (ICPD), 1994, which advocates that “Reproductive health care should include, among others: counseling, information, education, communication and prevention and the appropriate treatment of sterility.” There is consensus among researchers on the insufficient and precarious inclusion of low and high-complexity procedures in the SUS and the exclusion of same-sex couples and women and men without partners.

There is also discussion in the literature about intervention and medicalization of the female body based on the concern with reproductive health, given the high hormone dosages that women are subjected to in this process, which can be translated into the following question: how safe is Medically Assisted Reproduction and how far should we go to produce children? There is speculation that these techniques may be associated with an increased risk of long-term health problems for patients and children (Graham *et al*., 2023; Pandey *et al*., 2012; Schieve *et al*., 2004).

This discussion extends through the field of bioethics, reaching the genetic editing of embryos, the production of savior siblings^12^, embryo selection, and the creation of problems that can lead to such choices. Sex selection is one of them. Countries such as India and China have promoted gender discrimination and population imbalance based on female gender discrimination. Likewise, the possibilities that technologies already provide raise the option of choosing children with desirable characteristics. Many scholars fear this would be an open path to the practice of “eugenics.”

Recently, oocyte cryopreservation as a fertility preservation technique and cross-border oocyte and surrogate donation has been the main topics of investigation. There is a question about the effectiveness of oocyte cryopreservation in preserving female fecundity and carrying out future reproductive projects. However, the theme of “reproductive tourism” is the most discussed due to its complex intersection between gender, sexuality, class, and race/ethnicity. The present challenge is well stated by Sarojini *et al*. (2011): “How can we ensure that the crossing of geographical and ‘biological’ boundaries does not become a crossing of ethical boundaries?”

## CONCLUSION

Most policies for developing countries were centered on family planning and reducing fertility rates due to Malthusian thinking that provoked fears of a population explosion. For neo-Malthusians, birth control with contraceptive technologies would be the best way to reduce poverty in these countries with high population growth rates. Nowadays, we know that it is a myth. Infertility treatment is probably the most neglected and underestimated health problem in developing countries (Coutinho & Golgher, 2018; Ombelet, 2011).

In Brazil, we have declining fertility rates, but this combines a high proportion of unwanted fertility, a high relative fertility rate among 15-19-year-olds, and a trend toward an increased incidence of postponed motherhood. Coutinho & Golgher (2018) point out that the country has neglected infertility as an issue of reproductive rights. Recognizing the strong links between poverty and the presence of conditions that affect fertility, such as sexually transmitted infections, is a reproductive rights issue.

The challenge for the Brazilian State in the sexual and reproductive rights field is massive. Decades of profound demographic changes in a persistent scenario of social and economic inequalities have led to enormous reproductive injustice. Access to reproductive technologies remains exclusive and depends on insufficient State action to reach those who need it. The main barrier to access reproductive technologies in Brazil is economical, reproducing the same socio-economic inequalities associated with the prevalence of unplanned pregnancy. We also must consider the new designs of family configurations without neglecting the broader agenda of redeeming social debts.

The Brazilian contribution to the academic debate has been fruitful, although it still does not occupy the space that could stimulate new research in this field. There is an absolute lack of systematized data on the causes and prevalence of female and male infertility in Brazil and its social, economic, and psychological impacts. Data that characterize the affected population and the types of infertility are fundamental inputs for public policies. Also, in the field of data gaps, we have information on the knowledge of the reproductive cycle between women and men by sociodemographic characteristics, reproductive intention, gender negotiation processes, and the use of reproductive technologies in their multiple and varied circumstances - straight couples, women and men without partners, same-sex and transgender couples. There is also the need to investigate the supply and demand in Brazil for international services and their mercantile logic, including the various stakeholders’ views, motivations, and experiences.

Likewise, there is a need to produce data regarding the judicialization of assisted reproduction treatment through the SUS or health plans to comply with constitutional principles and the Family Planning Law of 1996. Moreover, it is of utmost importance to expand quantitative and qualitative studies on the phenomenon of assisted reproduction in Brazil to allow for an assessment of its scope and its social, demographic, and legal implications.

Finally, from the point of view of knowledge about the reproductive projects of specific population segments, it is imperative to produce quantitative data on infertility and unmet treatment needs, as well as qualitative data on perceptions and intentions to use reproductive technologies. Broadening and deepening the understanding of the phenomenon of assisted reproduction in Brazil and its social and demographic implications will contribute enormously to the strengthening of the Brazilian reproductive rights agenda.

## Footnotes

1 For a population to be entirely replaced, the fertility rate cannot be less than 2.1 children per woman.

2 DHS (Demographic Health Survey) is an investigation conducted worldwide with support from USAID (United States Agency for International Development) in partnership with several other international institutions. It aims to provide data and analysis for a broad set of planning, monitoring and impact assessment indicators in the areas of population, health and nutrition of women and children in developing countries.

3 Contracted services and procedures: fresh or frozen donor gametes, traditional IVF, IVF with ICSI (intracytoplasmic sperm injection), genetic testing, cryopreservation of oocytes and embryos, surrogate pregnancy.

4 BCTGs (Banks of Germ Cells and Tissues) have mandatory registration with ANVISA. They are responsible for selecting, collecting, transporting, recording, processing, storing, discarding, and releasing cells, germ tissues, and embryos, for their use or donation, of a public or private nature, for use in assisted reproduction.

5 Research funded by Fapesp - São Paulo Research Foundation (Process 2009/14981-8).

6 SisEmbrio - the National Embryo Production System was created by Anvisa Collegiate Board Resolution - RDC 29/2008 and updated by RDC 23/2011 (2011).

7 The import consent report granted by Anvisa is publicly accessible via the institution’s website. It provides information on the origin and destination of the samples by demographic region and state, as well as the main characteristics of gamete donors and the profile of sample applicants.

8 According to the publication The International Glossary on Infertility and Fertility Care (2017), PGT is defined as “A test performed to analyze the DNA from oocytes (polar bodies) or embryos (cleavage stage or blastocyst) for HLA-typing or for determination genetic abnormalities. These include: PGT for aneuploidies (PGT-A); PGT for monogenic/single gene defects (PGT-M); and PGT for chromosomal structural rearrangements (PGT-SR)” (Zegers-Hochschild et al., 2017).

9 The Brazilian Biosafety Law of 2005 - Law nº 11.105, of March 24, 2005 (2005) - criteria for research and therapy purposes using embryonic stem cells obtained from human embryos produced by in vitro fertilization. It prohibits any kind of genetic engineering.

10 There are genetic diseases that affect only one of the sexes. Only in these cases is sex selection allowed, to avoid the possibility of the same genetic alteration.

11 The main points of support of the Catholic religion against assisted reproduction are contained in the documents Evangelium Vitae and Instruction Donum Vitae. Among the various topics addressed, those of interest to the specific issue of reproduction are: 1) the beginning of life takes place in the act of conception and fertilization; 2) proper procreation can only occur through marriage between a man and a woman; 3) the scientific techniques used to solve infertility problems are legal, however, as long as they do not replace the sexual act; 4) couples unable to have children should adopt children; 5) abortion is considered one of the greatest sins of the Catholic Church and receives the most severe punishment in the Church, ex-communion.

12 The term savior siblings refer to children born with genetic characteristics explicitly chosen to treat a brother or sister’s illness. This possibility is already provided for in the CFM standard through Resolution 1.358/1992 (1992), which states that “AR techniques should not be applied with the intention of selecting the sex or any other biological characteristic of the future child, except when it comes to preventing diseases linked to the sex of the child to be born.” It also specifies that “any intervention on pre-embryos ‘in vitro,’ for diagnostic purposes, may have no other purpose than the assessment of its viability or detection of hereditary diseases, with the informed consent of the couple being mandatory”. However, there is no legislation in the Brazilian legal system.
